# Artificial intelligence-supported segmentation of cardiac anatomy in open-heart surgery videos

**DOI:** 10.1007/s11701-026-03775-x

**Published:** 2026-08-06

**Authors:** Maj Stenmark, Julia Önerud, Shahriar Anvariazar, Phan-Kiet Tran

**Affiliations:** 1 Pediatric Cardiac Surgery, Children’s Heart Center, Skåne University Hospital, Lund, Sweden; 2 Department of Clinical Sciences, Lund University, Sölvegatan 19, Lund, Sweden; 3 Department of Computer Science, Lund University, Lund, Sweden

**Keywords:** Artificial intelligence, Surgical decision-making, Anatomical segmentation, Open-heart surgery, SAM 2, YOLO

## Abstract

Accurate identification of anatomical structures is essential for safe congenital cardiac surgery and for the development of assistive robotic systems. However, scalable annotation of open-heart surgical video remains a major challenge due to dynamic tissue motion, occlusion, and anatomical variability. The aim of this study was to develop and evaluate a human-in-the-loop segmentation and tracking pipeline for congenital open-heart surgery videos. A dataset of 72 annotated video clips comprising 27,461 frames was created from routine recordings of congenital cardiac surgery. Independent tracking evaluation was performed on 6 video clips (2,400 frames) from three surgical cases that were not used for training or validation. A hybrid framework combining fine-tuned Segment Anything Model 2 (SAM 2) for propagation-based tracking and YOLOv11 for detection-based monitoring was implemented and evaluated. Fine-tuning improved temporal tracking performance compared with the pretrained model, increasing the mean IoU from 82.1% to 92.2% and the proportion of frames with IoU ≥ 90% from 48.2% to 72.3% on the independent evaluation dataset. A graphical user interface enabled efficient dataset creation, reducing annotation time by approximately 40-fold compared with manual tracing. This study demonstrates that domain-adapted foundation-model tracking can provide robust anatomical tracking in a dynamic open surgical environment and offers a scalable framework for future assistive and robotic cardiac applications.

## Introduction

Accurate recognition of anatomical structures is fundamental to safe surgical performance. Analysis of surgical adverse events has identified lack of recognition as a common human performance deficiency [1], underscoring the importance of reliable intraoperative visual interpretation. Recent advances in artificial intelligence (AI), particularly deep learning–based image analysis [2], have enabled automated object detection and segmentation with performance approaching or exceeding expert-level interpretation in selected medical domains [3, 4]. In surgery, AI-driven video analysis has been explored for workflow assessment, performance evaluation, and error detection [5–12], and mixed reality systems have shown promise for intraoperative support [13–15]. These developments are closely linked to emerging robotic and assistive surgical systems that rely on accurate visual scene understanding [16–18].

Despite rapid progress, most segmentation research in surgery has focused on laparoscopic procedures or preoperative imaging such as computed tomography [19–21]. Open congenital cardiac surgery presents distinct challenges. Cardiac structures exhibit substantial inter-patient variability in size, shape, and spatial relationships, and dynamic motion during the cardiac cycle introduces continuous deformation. Furthermore, occlusion by instruments and tissue manipulation complicates automated segmentation. The scarcity of annotated video data in this highly specialized domain remains a major obstacle to development of robust AI-supported systems.

Foundation segmentation models such as the Segment Anything Model (SAM) and its successor SAM 2 have demonstrated strong generalization capabilities across diverse visual domains [22]. In medical applications, several studies have shown that adapting foundation models improves segmentation performance, including early parameter-efficient adaptation of SAM (SAMed [23]), adaptation of SAM 2 to surgical videos (SurgiSAM2 [24]), and large-scale medical adaptation of SAM 2 across multiple imaging modalities (MedSAM2 [25]). Although general medical foundation models have also been proposed, recent work suggests that task-specific fine-tuning of the original foundation model can perform comparably to using a medically pretrained backbone, supporting adaptation from the original SAM 2 for specialized applications (MedicoSAM [26]). However, efficient and scalable workflows for generating high-quality annotated surgical video datasets remain underexplored, particularly in congenital cardiac surgery.

The aim of this study was to develop and evaluate an integrated human-in-the-loop framework for anatomical structure recognition in congenital open-heart surgery videos. By combining domain-adapted SAM 2 tracking, YOLO-assisted monitoring, and a custom graphical user interface for rapid annotation, correction, and validation, we sought to enable efficient dataset generation and robust propagation-based tracking of key cardiac structures. The system was evaluated with respect to segmentation accuracy, tracking stability, and annotation efficiency.

## Materials and methods

### Dataset

Video data were obtained from routine recordings at the Department of Pediatric Cardiac Surgery, Skåne University Hospital. The study was approved by the Swedish Ethical Review Authority (reference number 2021-05705-01). Written informed consent was obtained from patients’ legal guardians, and all data were anonymized prior to analysis.

Surgical procedures were recorded using two Sony FDR AX53 cameras mounted 105 cm above the surgical field. Videos were captured at 50 interlaced frames per second with resolutions of 1920 × 1080 and 1280 × 720 pixels. The institutional archive includes over 700 congenital cardiac procedures across approximately 40–50 different surgical types. Each surgical procedure corresponded to a unique patient, and no reoperations were included in the dataset. In total, the dataset contained 27,461 annotated frames. The SAM 2 model was fine-tuned on 65 clips (21,394 frames) from 40 surgical cases. Because YOLO was trained using the propagated segmentation masks as reference labels, only the subset of clips with the highest quality segmentations was used for YOLO training. The YOLO model was therefore trained on 31 clips (14,970 images) from 17 surgical cases, with 9 clips (2,687 frames) from 7 additional surgical cases used for validation. Primary evaluation was performed on 6 video clips (2,400 frames) from 3 independent surgical cases that were not used for training or validation. These clips were used to evaluate both SAM 2 tracking performance and YOLO detection performance. To evaluate long-term tracking and the effect of monitoring and reinitialization, two additional 30 s video clips from two further independent surgical cases were analyzed. Patient-level separation was enforced between the training, validation, and evaluation datasets. Clips were selected from the phase following sternotomy and prior to corrective intervention, when the heart was exposed and structures were clearly visible. Three anatomical structures were selected for segmentation based on size, visibility, and clinical relevance: the aorta, right atrium, and right ventricle.

### Segmentation pipeline

A semi-automatic, human-in-the-loop segmentation workflow combined YOLOv11 for object detection with SAM 2 for mask generation and video tracking.

**Fig. 1:**
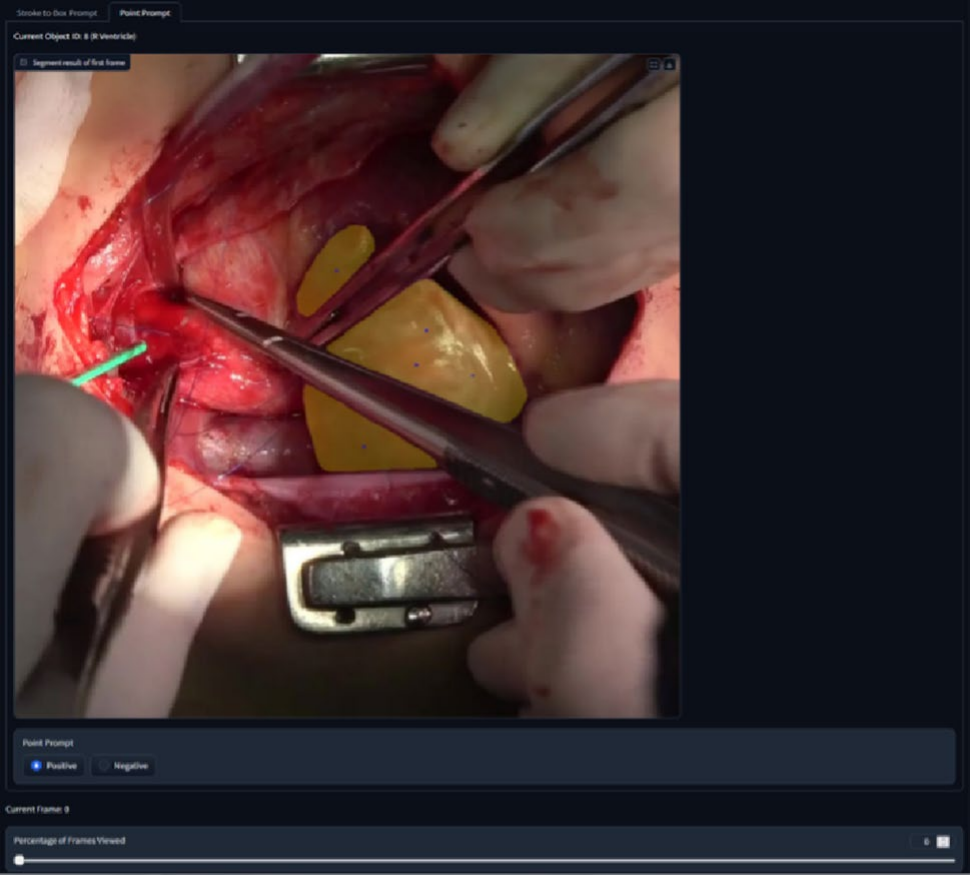
Graphical user interface for interactive segmentation. The user places positive point prompts (blue points) on the target anatomy, and the resulting segmentation mask is displayed as a yellow overlay for immediate visual assessment. The interface allows the user to review the propagated segmentation and iteratively add further prompts if correction is required

Initially, relevant 10-second video segments were manually identified and cropped to square format around the surgical field. In later iterations, a trained YOLO model was used to automatically detect heart structures and determine the optimal crop. For each clip, the user selected the target anatomy by placing one or more positive point prompts on the first frame through a custom graphical user interface (GUI; Fig. 1). Multiple positive prompts could be placed when parts of the anatomy were separated by surgical instruments or otherwise partially occluded. SAM 2 then generated segmentation masks for the selected structures and propagated them frame-by-frame throughout the clip.

The GUI allowed users to visualize, review, and correct the propagated segmentations. If errors were identified, additional positive or negative point prompts could be added to any frame before restarting propagation. This iterative refinement continued until satisfactory segmentation quality was achieved. Segmentation was primarily performed by an engineer using the GUI-assisted workflow. Whenever uncertainty arose regarding anatomical boundaries or image interpretation, a pediatric cardiac surgeon was consulted, and the segmentation was reviewed and corrected accordingly. No formal inter-rater annotation study was performed.

Final segmentation masks were stored and subsequently used to fine-tune both the SAM 2 and YOLO models, enabling progressive improvement of tracking performance. In the final configuration, YOLO predictions were used both for automatic initialization of segmentation and for monitoring tracking stability during propagation.

### Training details

The YOLOv11 segmentation model *yolo11l-seg* with 27.6 million parameters was trained on a workstation equipped with an NVIDIA RTX 4090 GPU. Fine-tuning of SAM 2 (SAM 2.1 Hiera Large, 224 million parameters) was performed on one NVIDIA A100 GPU. No validation-based early stopping was used for SAM 2. During training, SAM 2 maintains a streaming memory to keep temporally consistent segmentation. At 1024-pixel resolution, the memory stores 18 frames, whereas at 512-pixel resolution it stores 60 frames. Lower spatial resolution and frame rate allow the model to learn longer temporal dependencies. To balance temporal resolution and VRAM constraints, all training and inference clips were resampled to 40 fps and resized to square format. This choice was required to allow propagation-based tracking and fine-tuning to run within available VRAM on the RTX 4090 and A100 hardware. The training dataset used for SAM 2 was also augmented by resizing to 512-pixel resolution and frame-rate downsampling to 20, 10 and 5 fps. Other augmentation techniques provided by the official SAM 2 training code, such as horizontal flipping, were also used; see the code repository for the full configuration details. The model was trained using a fixed schedule without validation-based early stopping. The checkpoint with the lowest observed training loss (after 33 epochs) was selected for all subsequent experiments. The model was trained with a learning rate of 5 × 10^− 6^, a batch size of 1, and sigmoid focal loss. YOLO was trained with a learning rate of 0.01, a batch size of 10, multi-scale training, and early stopping based on validation performance. All inference experiments were performed at 1024-pixel resolution and 40 fps.

### Evaluation metrics

Segmentation accuracy was evaluated using Intersection over Union (IoU). YOLO detection performance was summarized at confidence thresholds of 50% and 85%. The 50% threshold represents a permissive operating point that maximizes detection coverage, whereas the 85% threshold was selected to represent high-confidence detections suitable for use as reliable reinitialization cues for SAM 2.

The fine-tuned model was evaluated against the pretrained model using two complementary metrics: the mean IoU for each heart structure and the percentage of frames with IoU ≥ 90%. The 90% threshold was selected a priori as a stringent criterion for consistently high segmentation accuracy. Statistical comparisons between methods were performed using the paired Wilcoxon signed-rank test. For each metric, values were first computed for each combination of video clip and heart structure, yielding 18 paired observations per comparison (6 video clips × 3 heart structures). The paired observations were matched by video clip and heart structure, and statistical significance was defined as *p* < 0.05.

Tracking performance of SAM 2 was evaluated under three conditions:

1. Continuous propagation without reinitialization.
2. Propagation with periodic monitoring and reinitialization from ground truth data.
3. Propagation with periodic monitoring and reinitialization from YOLO segmentation.

For monitoring, a custom congruence metric was applied every 8 frames to compare SAM 2 segmentations with YOLO detections. The congruence metric was intended to detect tracking drift while accounting for the dynamic nature of cardiac structures, where small spatial deviations may result in substantial IoU reductions despite clinically acceptable alignment.

The congruence metric was designed as a heuristic measure for detecting tracking failures rather than as an optimized similarity metric. High-confidence YOLO detections were emphasized through nonlinear confidence weighting because they provide more reliable reference segmentations. Recall similarity was weighted slightly more strongly than precision similarity to bias the metric toward detecting potential under-segmentation. The nonlinear exponents were introduced to increase sensitivity to larger disagreements while reducing sensitivity to minor boundary variations. The parameters were selected empirically during development and were not formally optimized.

The congruence metric combines:

- YOLO detection confidence ($\:{C}_{total}$),
- Recall similarity ($\:{S}_{A}$) representing under-segmentation of SAM 2 relative to YOLO,
- Precision similarity ($\:{S}_{B}$) representing over-segmentation of SAM 2 relative to YOLO.

Congruence was calculated as:

$$\:K=\frac{{\left({C}_{total}\right)}^{1.5}}{0.9}\left(\frac{3{\left({S}_{A}\right)}^{1.2}+2{\left({S}_{B}\right)}^{1.2}}{5}-0.9\right)$$

Where $\:{C}_{total}$ is the YOLO prediction confidence, with a nonlinear weighting emphasizing high-confidence detections. $\:{S}_{A}$ denotes the fraction of high-confidence YOLO segmentation intersecting with SAM 2 segmentation, and $\:{S}_{B}$ denotes the fraction of SAM 2 segmentation intersecting with YOLO segmentation. *K* is normalized such that values close to 1.0 indicate strong agreement; values below 0.9 were defined as unacceptable and triggered reinitialization.

Congruence penalizes both under- and over-segmentation, with greater weighting assigned to high-confidence YOLO detections. A threshold of 90% congruence was defined as acceptable tracking agreement. If congruence fell below this threshold for any structure, tracking was reinitialized using updated segmentation input before continuing propagation.

**Fig. 2:**
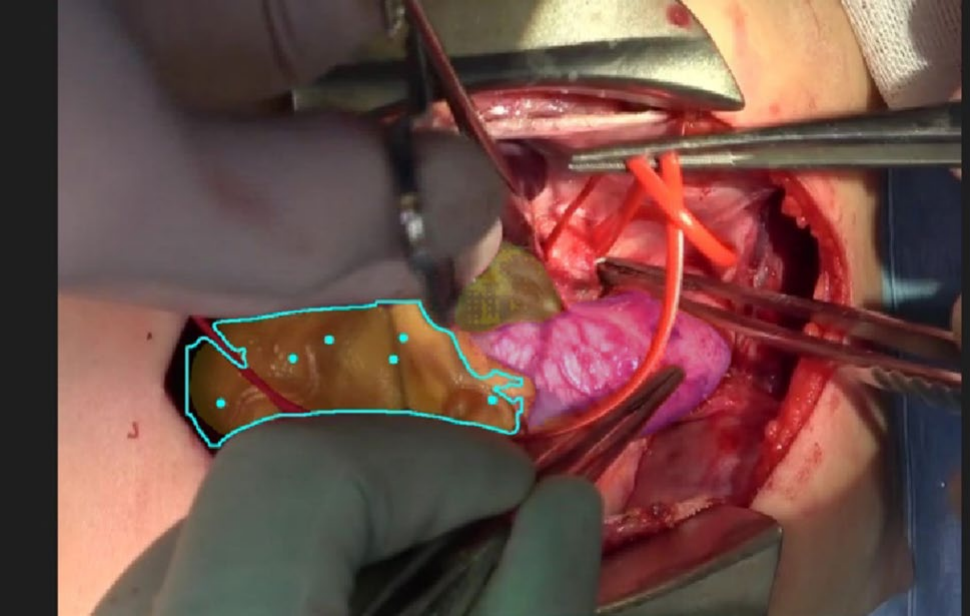
Example of the point sampling strategy used for reinitialization. Positive point prompts are sampled randomly from within the segmentation mask near the object boundary. For clarity, only six sampled points are shown

Congruence was calculated per structure. The SAM 2 version used in this study did not allow reinitialization of the segmentation mask for only one object, instead it required all structures to be reinitialized. The tracking accuracy was generally high, so instead of reinitializing all structures when one was failing, potentially causing poorer tracking overall, the monitoring and reinitialization was parallelized to only monitor and reinitialize one structure at a time. The reinitialization was evaluated using sample points from either the ground truth data (condition 2) or from YOLO segmentations (condition 3). For reinitialization, 20 positive point prompts were sampled randomly from within the segmentation mask, with sampling restricted to a region adjacent to the object boundary (Fig. 2). Restricting the sampling to the mask ensured that all prompts were positive, while concentrating them near the boundary provided information about the full spatial extent of the structure.

Annotation efficiency was assessed by comparing manual frame-by-frame segmentation to segmentation performed using the GUI. Time per frame was calculated for both approaches.

## Results

### Fine-tuned SAM 2 segmentation accuracy

**Fig. 3:**
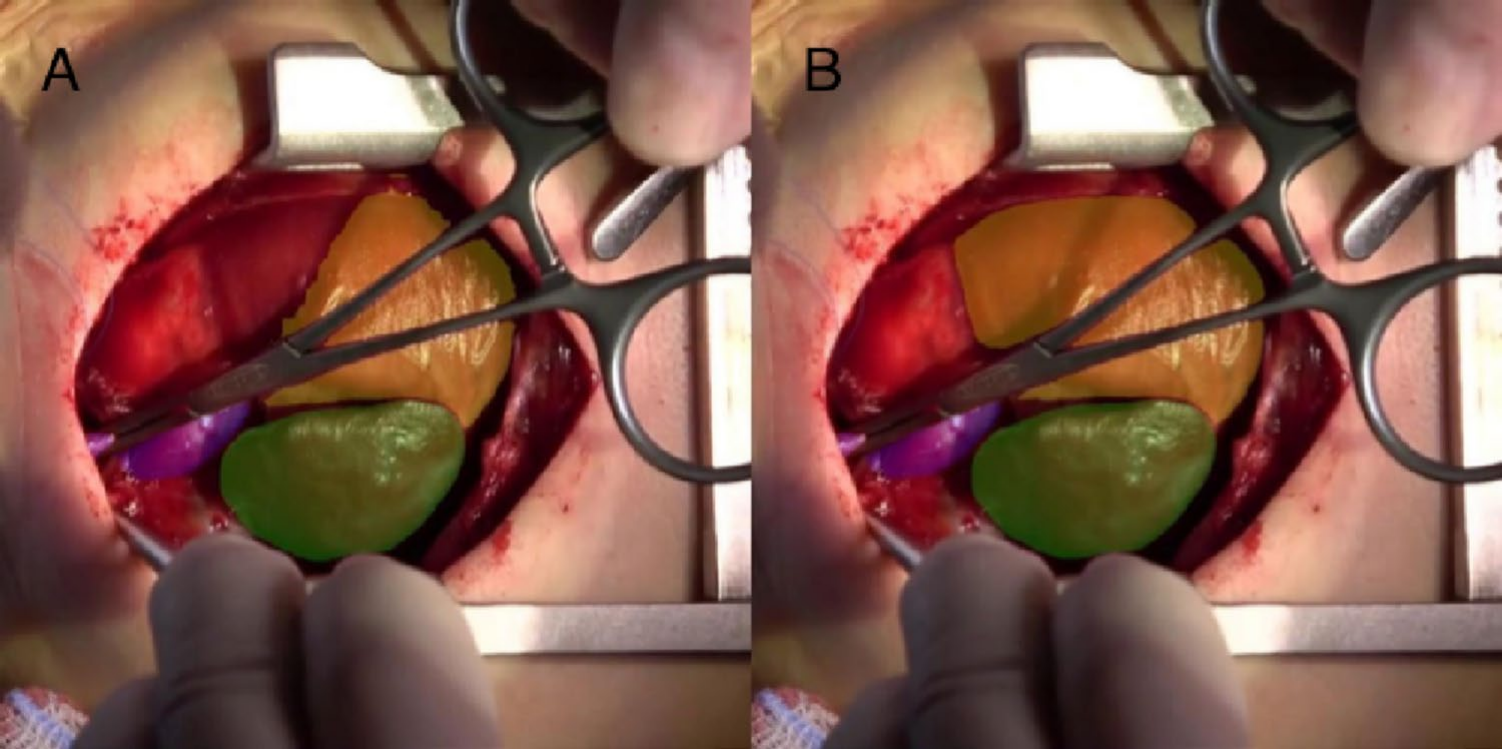
Qualitative comparison of segmentation tracking using the pretrained (**A**) and fine-tuned (**B**) SAM 2 models. The aorta is shown in purple, the right atrium in green, and the right ventricle in orange. The pretrained model exhibits tracking drift resulting in under-segmentation of the right ventricle, whereas the fine-tuned model maintains accurate segmentation throughout the sequence

**Fig. 4:**
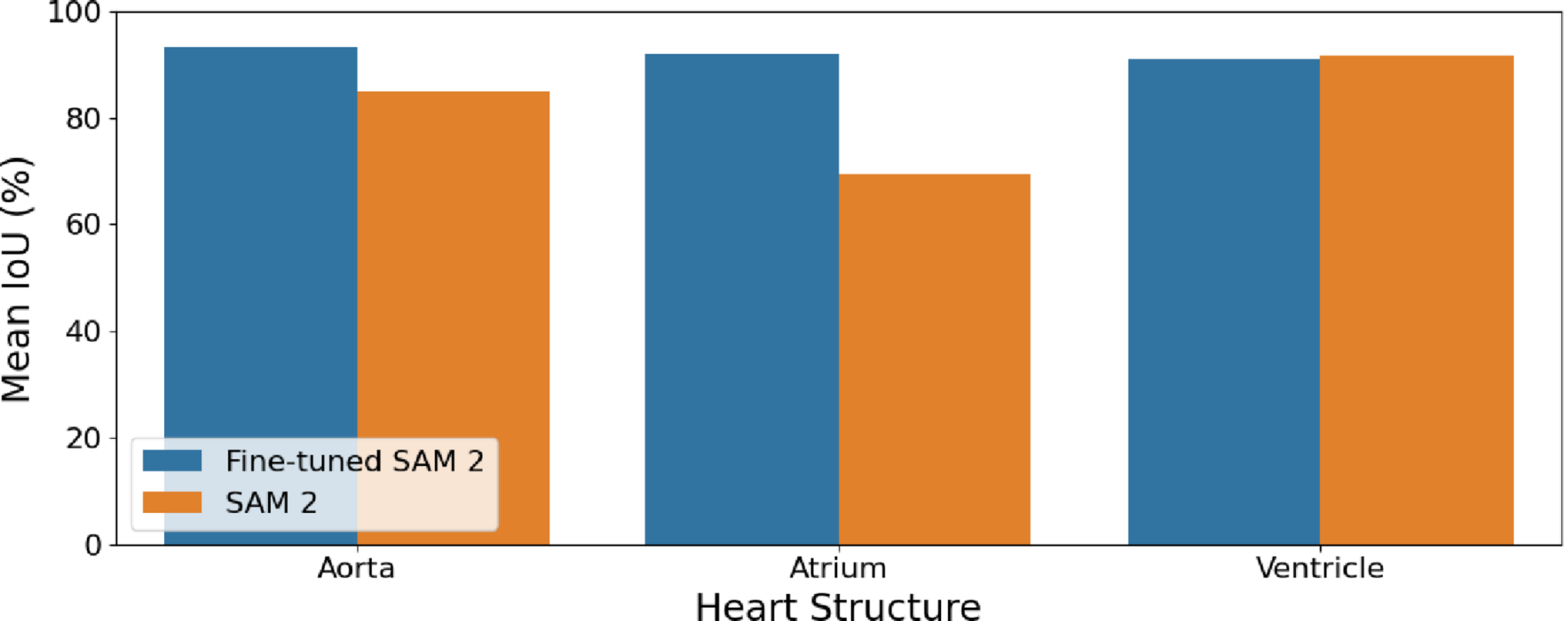
Mean Intersection over Union (IoU) for the fine-tuned and pretrained SAM 2 models across the evaluated heart structures

When initialized with the pretrained SAM 2 weights, the model typically maintained visually correct masks for only short intervals before tracking drift occurred (commonly after 1–2 s of continuous propagation), especially when instruments or hands partially occluded the field (Fig. 3A). After fine-tuning SAM 2 on the surgical dataset (see Training details), temporal stability of propagation improved markedly: the fine-tuned model maintained alignment with the visible anatomy for substantially longer segments than the untrained model before drift onset (see Fig. 3B). The pretrained SAM 2 performed poorly on the aorta and atrium, but well on the ventricle (see Fig. 4).

After 33 epochs of fine-tuning, the SAM 2 model had a Dice loss of 0.029 at 512 resolution and 0.035 at 1024 resolution, with a mean sigmoid focal loss of 0.001 and mean IoU loss of 0.012. The segmentation accuracy during tracking was evaluated on 2,400 frames from 6 video clips extracted from 3 independent surgical cases (10 s per clip; described in the Methods).

**Fig. 5:**
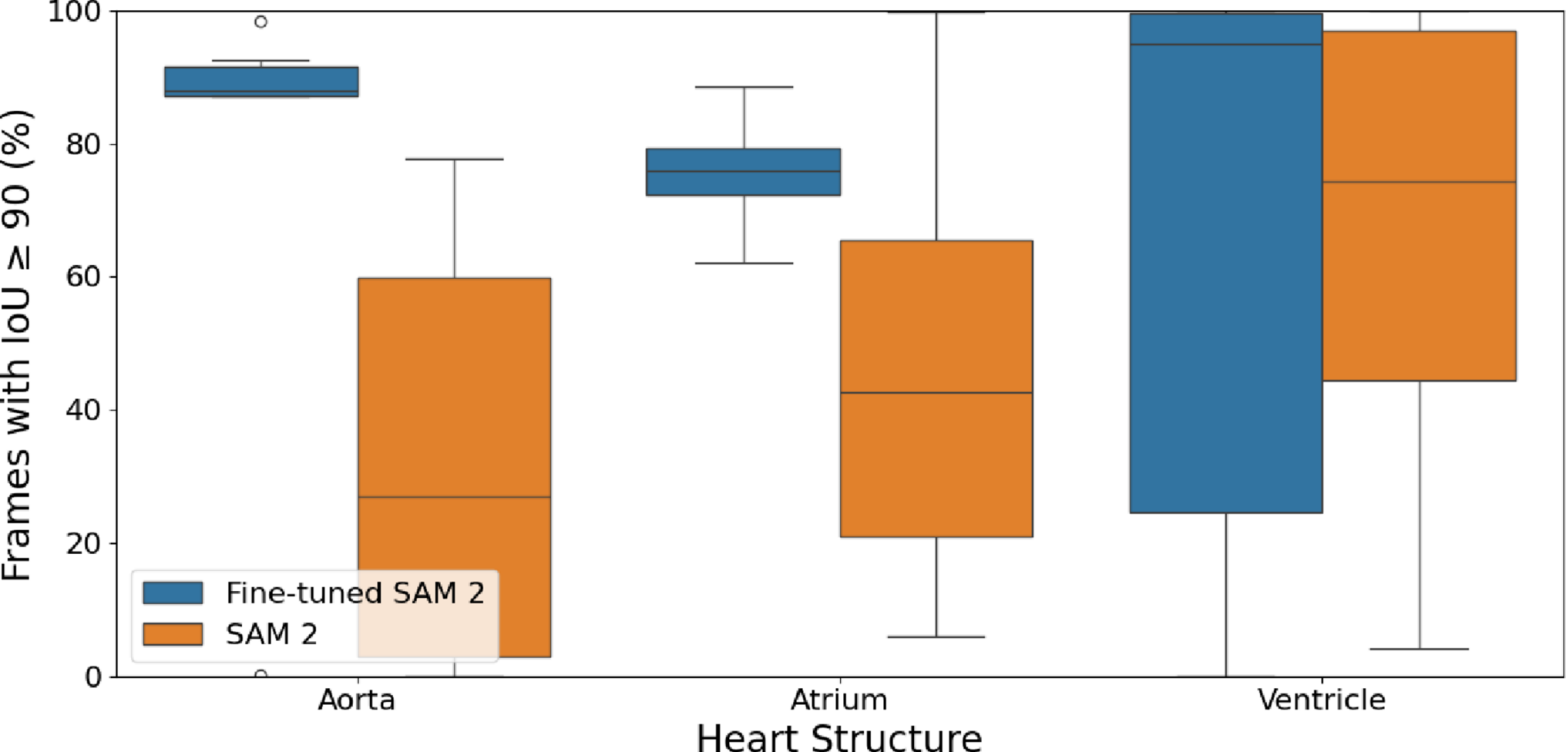
Percentage of frames with IoU ≥ 90% for the fine-tuned and pretrained SAM 2 models across the evaluated heart structures

As shown in Fig. 4, fine-tuning improved segmentation performance for the aorta and atrium, whereas performance for the ventricle was comparable to that of the pretrained model. Overall, the mean IoU increased from 82.1 ± 20.7% to 92.2 ± 6.5% (Wilcoxon signed-rank test, *W* = 30, *p* = 0.01). Figure 5 shows the percentage of frames with IoU ≥ 90% for each heart structure. Fine-tuning increased the proportion of frames achieving this threshold from 48.2 ± 36.6% to 72.3 ± 34.4%, and the improvement was statistically significant (Wilcoxon signed-rank test, *W* = 35, *p* = 0.049).

### YOLO accuracy

**Fig. 6:**
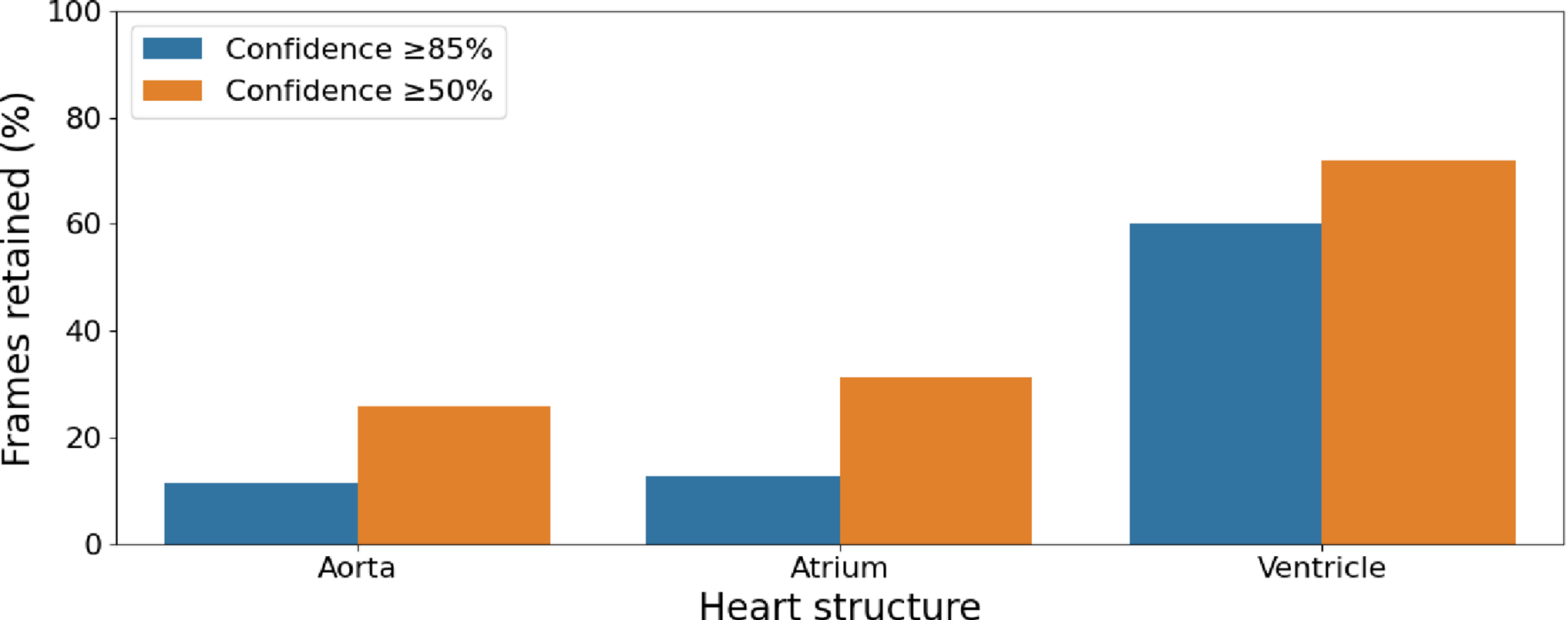
Percentage of frames retained by YOLO at confidence thresholds of 50% and 85% for each cardiac structure. Increasing the confidence threshold reduces the proportion of accepted detections across all structures

**Fig. 7:**
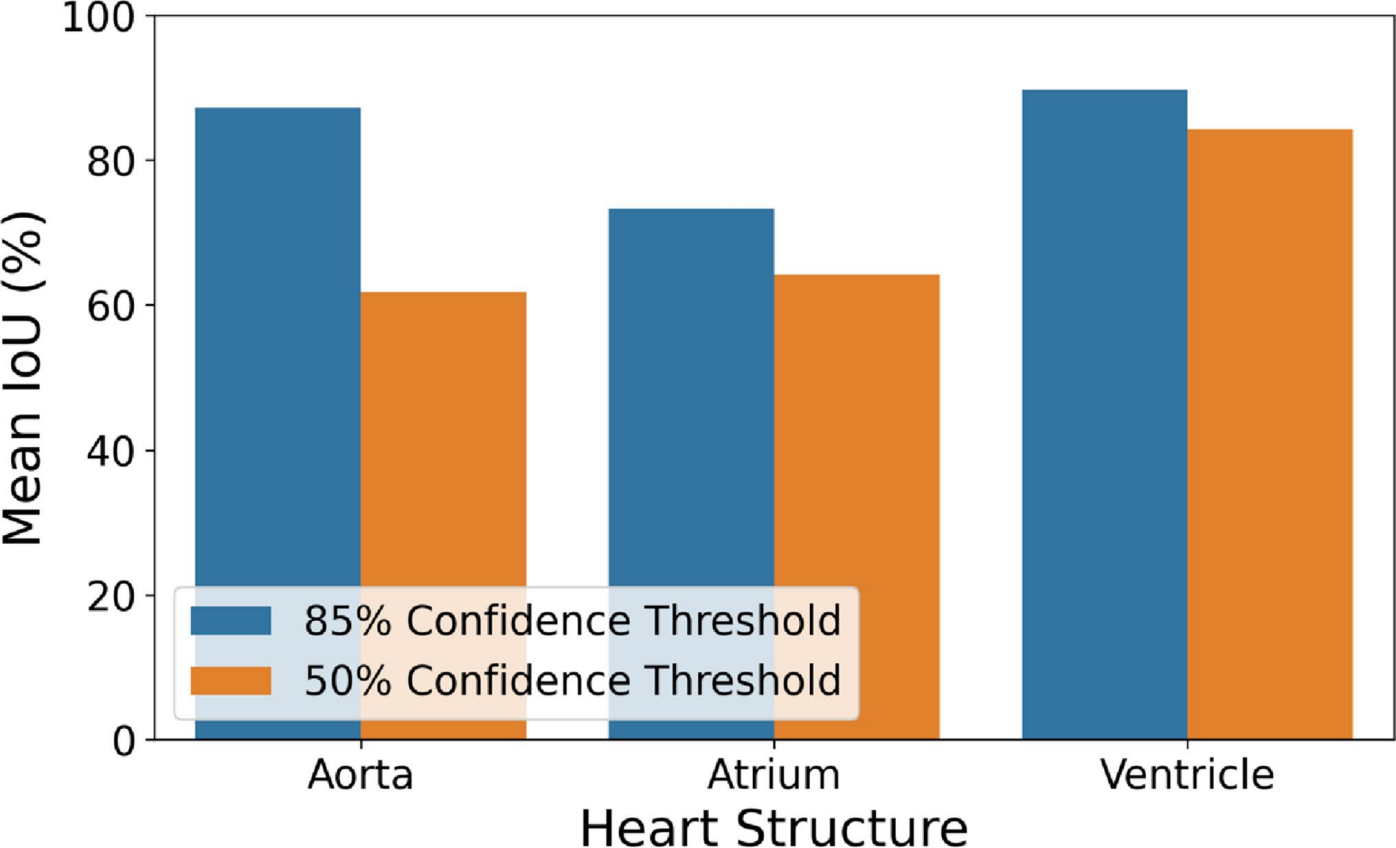
Mean IoU of retained YOLO detections at confidence thresholds of 50% and 85% for each cardiac structure. Mean IoU was calculated only for frames in which a detection was retained

YOLO detection performance was evaluated on the independent tracking evaluation dataset (2,400 frames; see Dataset). The effect of the confidence threshold on detection coverage and localization accuracy was evaluated (Figs. 6 and 7). At a confidence threshold of 85%, the ventricle was retained in 60.2% of frames, whereas the atrium and aorta were retained in only 12.7% and 11.4% of frames, respectively (Fig. 6). Lowering the confidence threshold to 50% increased the proportion of retained frames for all structures, from 11.4% to 25.8% for the aorta, from 12.7% to 31.2% for the atrium, and from 60.2% to 72.0% for the ventricle.

Increasing the confidence threshold improved the localization accuracy of the retained detections (Fig. 7). The mean IoU increased from 61.8% to 87.2% for the aorta, from 64.2% to 73.4% for the atrium, and from 84.3% to 89.8% for the ventricle when the confidence threshold was increased from 50% to 85%. These results demonstrate the expected trade-off between detection coverage and localization accuracy: a lower confidence threshold retains more detections, whereas a higher confidence threshold yields fewer but more accurate detections.

### Monitoring and reinitialization

**Fig. 8:**
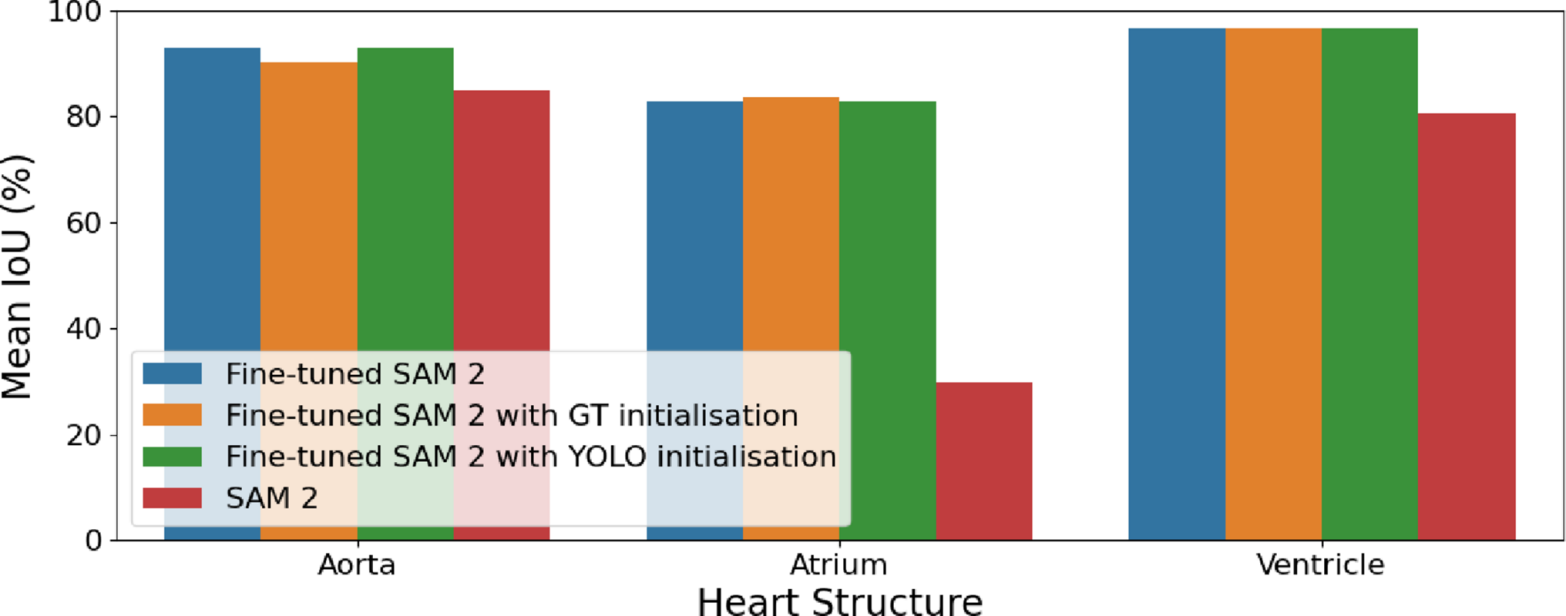
Mean Intersection over Union (IoU) for two independent 30 s evaluation videos. Results are shown for the fine-tuned SAM 2 model with continuous propagation (blue), the fine-tuned model with periodic reinitialization from ground-truth segmentations (orange), the fine-tuned model with periodic reinitialization from YOLO segmentations (green), and the pretrained SAM 2 model (red). Reinitialization did not improve tracking performance compared with continuous propagation

**Fig. 9:**
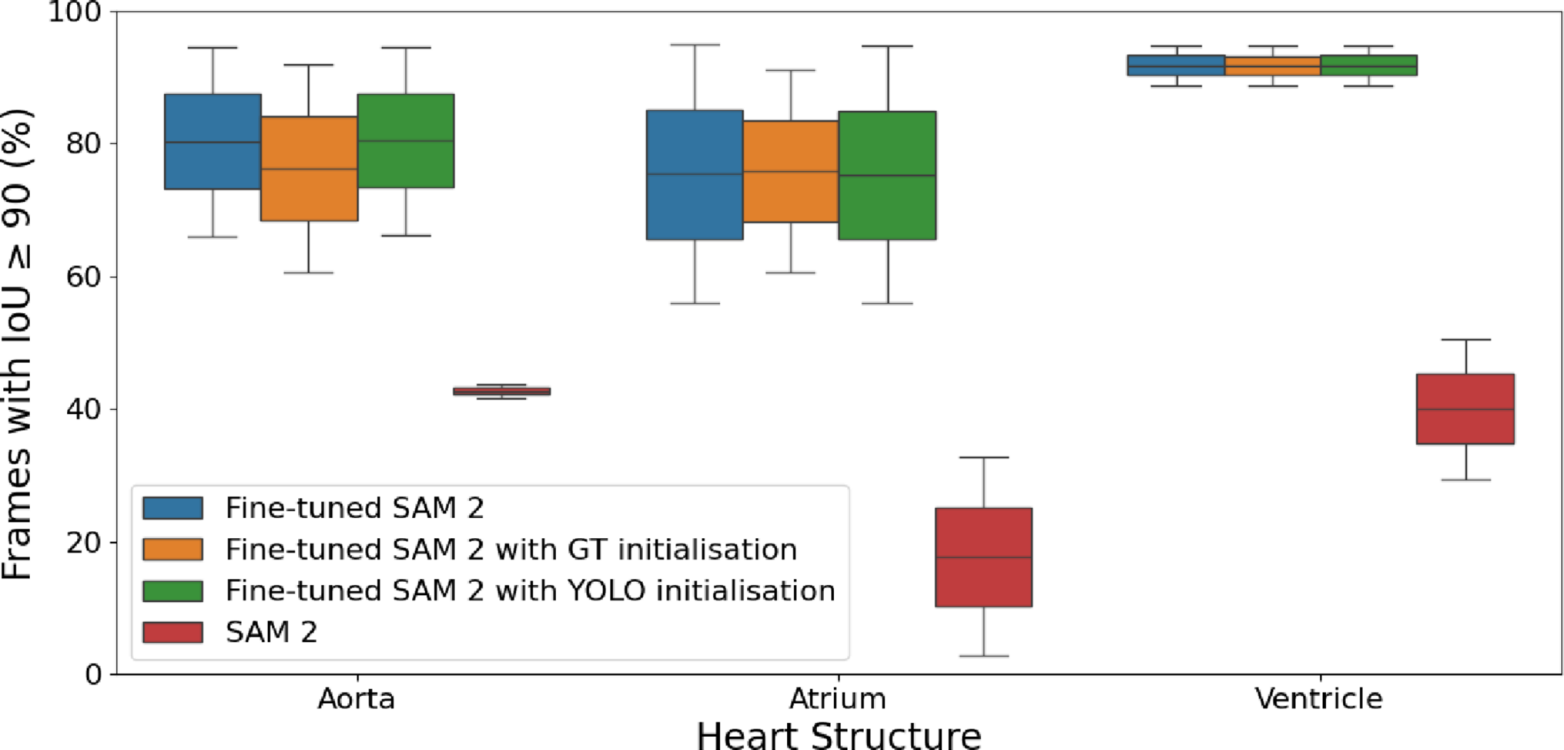
Percentage of frames with IoU ≥ 90% for the two independent 30 s evaluation videos. Results are shown for the fine-tuned SAM 2 model with continuous propagation (blue), the fine-tuned model with periodic reinitialization from ground-truth segmentations (orange), the fine-tuned model with periodic reinitialization from YOLO segmentations (green), and the pretrained SAM 2 model (red). The fine-tuned model maintained consistently high tracking performance regardless of reinitialization strategy

For the 10 s evaluation clips, tracking drift in the fine-tuned model was limited, and periodic monitoring with reinitialization did not measurably improve segmentation performance. Similar results were obtained for the two independent 30 s evaluation videos (Figs. 8 and 9). The fine-tuned model achieved substantially higher tracking accuracy than the pretrained model, whereas reinitialization from either ground-truth segmentations or YOLO segmentations produced little or no additional improvement.

As shown in Fig. 8, the mean IoU remained similar across all three fine-tuned configurations, indicating that continuous propagation was generally sufficient over the evaluated sequences. Likewise, Fig. 9 shows that the percentage of frames with IoU ≥ 90% was comparable for the fine-tuned model with and without reinitialization.

In the first video, the fine-tuned SAM 2 model maintained stable tracking throughout the sequence, leaving little opportunity for reinitialization to improve performance. In the second video, the atrium repeatedly entered and exited the field of view, reducing the performance of both SAM 2 and YOLO. Despite these challenging conditions, periodic reinitialization still did not provide a measurable improvement over continuous propagation.

### Annotation efficiency

To evaluate annotation efficiency, a cardiac surgeon segmented the same video clip using two approaches: manual frame-by-frame tracing and the graphical user interface (GUI)-assisted workflow.

Manual segmentation of 9 frames required 25 min and 28 s (163 s per frame). Using the GUI, segmentation of a 391-frame video clip required 11 min and 57 s (1.8 s per frame) to achieve acceptable segmentation quality. To reach segmentation quality comparable to careful manual tracing, additional corrective prompts were required approximately once every 70 frames, corresponding to an estimated 3.8 s per frame.

In this proof-of-concept timing experiment, the GUI-assisted workflow required approximately a 40-fold reduction in annotation time.

The full dataset of 27,461 frames was subsequently annotated using the GUI-assisted workflow, requiring approximately 30 h in total (~ 4 s per frame). These results demonstrate substantial efficiency gains when combining propagation-based segmentation with targeted human correction.

The timing measurements should be interpreted as an illustrative comparison of the annotation workflows rather than a strictly controlled benchmark, as the GUI-assisted workflow exploits temporal propagation whereas manual annotation requires independent frame-by-frame tracing.

## Discussion

This study demonstrates that a foundation-model–based segmentation pipeline can be successfully adapted for anatomical tracking in congenital open-heart surgery. The combination of domain-adapted SAM 2, interactive human-in-the-loop annotation, and propagation-based tracking enabled efficient creation of a large annotated surgical video dataset while improving temporal tracking stability. Together, these results demonstrate the feasibility of propagation-based segmentation in a challenging open surgical environment.

Recent studies have demonstrated that adapting foundation segmentation models to medical domains substantially improves segmentation performance compared with pretrained models. SurgiSAM2 [24] demonstrated that fine-tuning SAM 2 improves anatomical segmentation in laparoscopic surgery, while MedSAM2 [25] reported similar improvements across diverse medical imaging modalities, including endoscopy and echocardiography. Our findings are consistent with these studies and extend foundation-model–based segmentation to congenital open-heart surgery, which, to our knowledge, has not previously been investigated using foundation segmentation models.

Whereas MedSAM2 primarily focuses on developing a general-purpose foundation model for multiple medical imaging modalities, the present study addresses a different challenge: adapting an existing foundation model to enable long-term temporal propagation and efficient annotation in congenital open-heart surgery videos. Rather than proposing a new segmentation architecture, we demonstrate how domain-specific fine-tuning, interactive point prompting, and propagation-based tracking can be integrated into a practical annotation workflow for this application.

This focus is motivated by the unique challenges of congenital open-heart surgery. Cardiac structures vary considerably between patients, undergo continuous deformation during the cardiac cycle, and are frequently partially occluded by surgical instruments and tissue manipulation. These characteristics make robust long-term propagation substantially more challenging than in many previously studied surgical and medical imaging applications.

The GUI-assisted workflow also complements previous work on interactive annotation. MedSAM2 reported an approximately 85% reduction in annotation effort across multiple public medical datasets using propagation-assisted annotation. Direct numerical comparison between the studies is difficult because of substantial differences in imaging modalities, anatomical complexity, annotation protocols, and video duration. Nevertheless, our workflow likewise demonstrated a substantial reduction in annotation effort, reducing annotation time by approximately 40-fold and enabling efficient generation of more than 27,000 annotated video frames in a clinically challenging surgical domain. Scalable annotation strategies are essential for developing AI systems in highly specialized surgical domains where expert annotation time is limited. Although the GUI was designed to simplify the annotation process, accurate annotation still depended on anatomical expertise, highlighting the importance of clinician involvement in creating high-quality reference datasets.

The main contribution of this study is therefore not the observation that fine-tuning improves foundation-model performance, which has been demonstrated previously, but the development and evaluation of an integrated framework for congenital open-heart surgery. The proposed pipeline combines domain-adapted SAM 2 tracking, GUI-assisted interactive annotation, and YOLO-based monitoring within a workflow designed for scalable dataset creation and robust temporal tracking.

The congruence-based reinitialization strategy addressed a common limitation of propagation-based trackers: gradual drift over time. By incorporating YOLO detections as periodic anchors, the system was able to detect divergence and reset tracking when necessary. However, the present evaluation included 10 s clips and two 30 s videos, in which tracking drift of the fine-tuned model was generally limited. Consequently, these results should not be interpreted as evidence that reinitialization is unnecessary. Instead, they indicate that no measurable benefit was observed under the evaluated conditions. Several factors likely contributed to the absence of measurable improvement, including the high tracking stability of the fine-tuned model over the evaluated video lengths and the limited availability of high-confidence YOLO detections for reinitialization. Longer video sequences are likely to provide a more appropriate setting for evaluating the potential benefits of drift monitoring and automatic reinitialization.

Fine-tuning was performed offline using high-memory GPU hardware, whereas deployment would require only model inference. Inference latency and real-time performance were not evaluated in the present study but represent important considerations for future intraoperative implementation.

From a clinical perspective, automated tracking of major cardiac structures may contribute to future assistive systems in open surgery by providing continuous anatomical awareness despite tissue motion and instrument occlusion. Such information could support visual guidance and surgical scene understanding, both of which are considered important prerequisites for future context-aware assistive and robotic systems. However, anatomical segmentation and scene understanding remain largely research topics and are not yet routinely integrated into clinical surgical workflows. The present work should therefore be viewed as a technical proof-of-concept addressing one of the enabling technologies for future AI-assisted and robotic surgery. While the present study does not evaluate real-time deployment during surgery, the results provide a technical foundation for further translational development.

## Limitations

### This study has several limitations

First, the dataset was obtained from a single surgical center using a fixed camera configuration. In addition, the independent evaluation was performed on a limited number of surgical cases. Although multiple clips and anatomical structures were included in the evaluation, larger multicenter validation studies are needed to establish robustness across broader anatomical variation, surgical procedures, recording conditions, and institutions. Consequently, the reported segmentation and tracking performance should be interpreted as proof-of-concept rather than as a definitive assessment of generalizability across diverse surgical settings.

Second, only three large external cardiac structures, the aorta, right atrium, and right ventricle, were included. Smaller or intracardiac structures may present greater segmentation and tracking challenges due to reduced visibility, complex morphology, or occlusion.

Third, reference segmentations were generated using a semi-automatic, human-in-the-loop workflow rather than independent multi-expert manual annotation. Although uncertain cases were reviewed together with a pediatric cardiac surgeon during dataset creation, no formal assessment of inter-rater agreement, intra-rater repeatability, or annotation uncertainty was performed. Consequently, some reference-standard bias may remain in the training and evaluation data.

Fourth, YOLO detection coverage varied substantially between anatomical structures, influencing the frequency and effectiveness of congruence-based reinitialization.

Fifth, the congruence metric was manually designed, and its parameters were selected empirically. A systematic optimization and sensitivity analysis of the weighting coefficients, nonlinear exponents, and decision threshold was beyond the scope of this study and should be addressed in future work.

Finally, annotation efficiency was evaluated in a proof-of-concept timing experiment performed by a single cardiac surgeon. Although the observed reduction in annotation time was substantial, repeated measurements across multiple annotators and datasets would be required to characterize the variability and reproducibility of the workflow. Furthermore, the present study evaluated offline tracking performance and annotation efficiency. Real-time performance, latency, and integration into robotic or intraoperative systems were not assessed and remain subjects for future investigation.

## Conclusions

This study demonstrates that a foundation-model–based approach can be adapted for anatomical tracking in congenital open-heart surgery using an iterative human-in-the-loop workflow. Fine-tuning of SAM 2 substantially improved temporal tracking stability compared to the pretrained model. The graphical user interface reduced annotation time by approximately 40-fold compared to manual tracing, facilitating scalable dataset generation in a highly specialized surgical domain.

## Data Availability

The segmentation data will be made public upon publication.
